# Supplementary figures and images for: Genome-wide discovery and characterization of long noncoding RNAs in African oil palm (Elaeis guineensis Jacq.)
Source: PeerJ. 2020 Nov 2;8:e9585. doi: 10.7717/peerj.9585 (PMC7643553; doi:10.7717/peerj.9585)

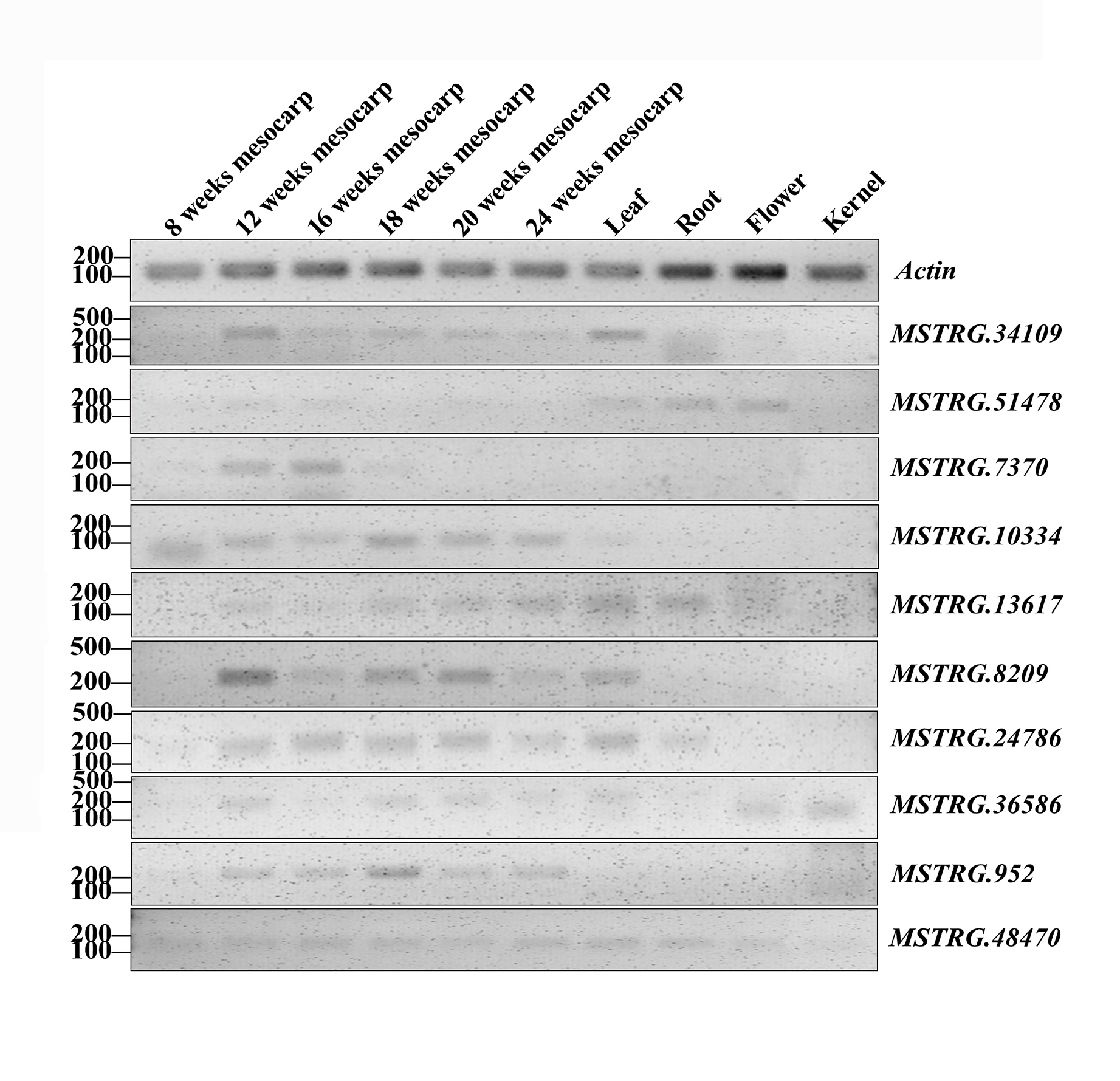

Supplement: Supplemental Information 10 [file peerj-08-9585-s010.jpg]
